# An innovative reconstruction procedure for fractures extending to the posterior orbital floor: utilizing the inferior margin of the greater wing of the sphenoid bone for reconstruction

**DOI:** 10.1007/s00405-024-08808-5

**Published:** 2024-07-31

**Authors:** Kosuke Takabayashi, Yohei Maeda, Nobuya Kataoka

**Affiliations:** 1 Department of Otorhinolaryngology, Japanese Red Cross Asahikawa Hospital, Asahikawa City, Hokkaido Japan; 2 Department of Otorhinolaryngology, Sapporo Medical University School of Medicine, Sapporo City, Hokkaido Japan; 3 Department of Otorhinolaryngology, Japan Community Health Care Organization Osaka Hospital, 4-2-78 Fukushima, Fukushima-ku, Osaka City, Osaka 553-0003 Japan; 4 Department of Otorhinolaryngology, Head and Neck Surgery, Osaka University Graduate School of Medicine, Suita City, Osaka Japan; 5 Department of Ophthalmology, Japanese Red Cross Asahikawa Hospital, Asahikawa City , Hokkaido Japan

**Keywords:** Greater wing of the sphenoid bone, Infraorbital nerve, Superior posterior wall of the maxillary sinus, Transorbital approach, Transnasal approach

## Abstract

**Purpose:**

No definitive procedures have been proposed for orbital floor fractures extending to the slope of the posterior end, which is a challenging problem. This study demonstrates the effectiveness of an orbital reconstruction procedure based on anatomical landmarks that we developed, called the three landmarks procedure (TLP).

**Methods:**

This study is a single-center retrospective cohort study conducted by the Department of Otorhinolaryngology, Japanese Red Cross Asahikawa Hospital. Data were collected from April 2000 to December 2023. The effect of TLP and the balloon procedure (BP) on ocular movement was compared. The prevalence of postoperative enophthalmos after TLP was examined.

**Results:**

The study included 17 patients who underwent TLP and 25 patients who underwent BP. Postoperative mean Hess area ratio (HAR%) was 98.3 (95% confidence interval (CI), 97.0–99.6) in the TLP group and 88.6 (95% CI 83.2–94.0) in the BP group. Among study patients with fractures extending to the posterior slope, 14 underwent TLP and 16 underwent BP. Postoperative mean HAR% was 98.5 (95% CI 97.3–99.7) in the TLP group and 89.2 (95% CI 82.4–95.8) in the BP group. Among all patients who underwent TLP, mean postoperative enophthalmos was 0.06 mm (95% CI − 0.32 to 0.44). It was 0.14 mm (95% CI − 0.31 to 0.59) among patients with fractures extending to the posterior slope.

**Conclusion:**

TLP resulted in better postoperative ocular movements than BP. Furthermore, TLP is an effective technique for treating fractures extending to the posterior slope, which are challenging to reconstruct.

## Introduction

In orbital floor reconstruction, fractures involving the slope of the posterior end of the orbital floor make it difficult to recognize the posterior fracture margin and to determine the best location for implant placement [1–3]. Surgical techniques have improved with the use of endoscopes, which allow the surgical field at the posterior end of the orbital floor to be clearly recognized [1, 2, 4], and navigation systems, which allow for accurate identification of the fracture’s location [5]. On the other hand, it has been reported that good results can be obtained using an endoscopic transnasal approach, in which a balloon is implanted in the maxillary sinus for repair and fixation [6–8]. Recognizing the anatomic landmarks for reconstruction is important for reproducible orbital reconstruction. The infraorbital nerve is a landmark that follows the orbital floor anterior to the pterygopalatine fossa [9]. The superior posterior wall of the maxillary sinus does not deviate, even when there is a fracture in the orbital floor [10]. Thus, it is a reliable landmark for the placement of a plate for orbital floor reconstruction.

Fractures that extend to the slope of the posterior end of the orbital floor break the site of implant placement at the posterior fracture margin [1, 2]. Therefore, reconstruction has been difficult because there is no space to place implants. Even with the endoscopic transnasal approach, a fracture at the posterior end is not completely repaired given the shape of the balloon. Furthermore, since there have been no landmarks connecting the infraorbital nerve to the superior posterior wall of the maxillary sinus, it was difficult to safely identify the deepest landmarks.

In this study, we examined the effectiveness of a technique we previously reported, which we named the three landmarks procedure (TLP), in which the inferior margin of the greater wing of the sphenoid bone is reconstructed as a new landmark [11]. In addition to evaluating the surgical outcomes of patients who underwent TLP and balloon fixation via an endoscopic transnasal approach, we compared the surgical outcomes of the two techniques, focusing on fractures extending to the slope of the posterior end of the orbital floor.

## Methods

### Study design and setting

This retrospective single-center cohort study was conducted by the Department of Otorhinolaryngology, Japanese Red Cross Asahikawa Hospital. Data were collected from April 2000 to December 2023.

### Participants

The study population consisted of patients with orbital floor fractures, except for the linear type, who underwent surgical treatment. They were divided into two groups by surgical procedure: TLP [11] or balloon procedure (BP) [6–8]. Inclusion criteria were orbital floor fracture and ability to follow the patient until fixation of ocular movement after surgery. Exclusion criteria were surgical procedure other than TLP and BP, lack of follow-up until fixation of ocular movement, and missing data on variables such as Hess area ratio (HAR%), fracture area, or fracture type.

### Surgical techniques

#### TLP

**Fig. 1:**
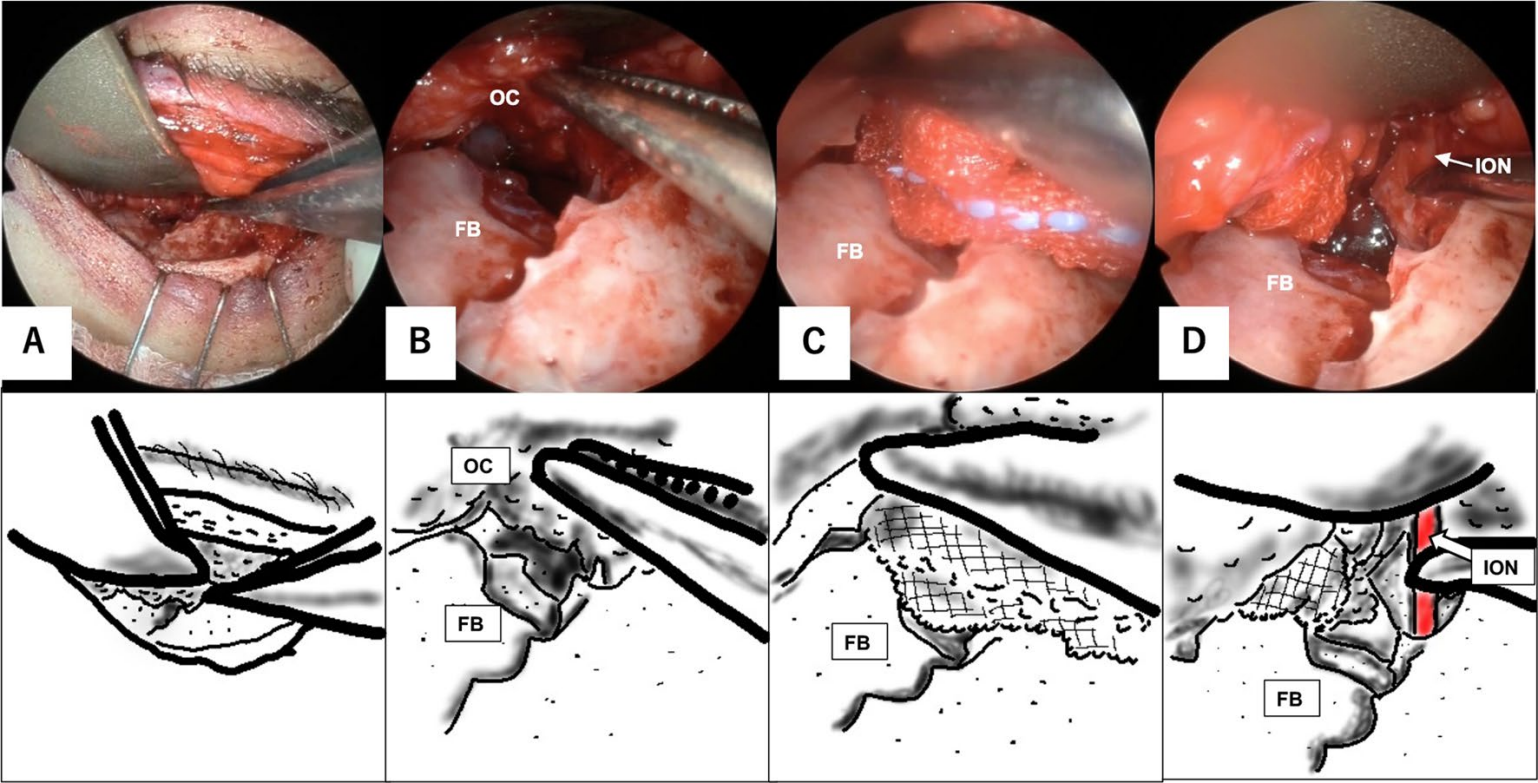
Stepwise view of the three landmarks procedure via the transorbital approach. Each endoscopic image is accompanied by a corresponding illustration below. The three landmarks are shown in red. Image **A** is a view of the fractured orbital floor via the transorbital approach with a subciliary incision. The orbital contents are elevated under the periosteum as much as possible. In this case, orbital fat was identified around the fractured area due to laceration of the periosteum (**B**). Although as much of the circumference of the fractured edge as possible is identified, the posterior area is difficult to identify due to the orbital contents (**C**). After the infraorbital nerve is identified, go posterior with elevation of the orbital contents at the superior margins of the infraorbital nerve (**D**). *FB* fractured bone, *ION* infraorbital nerve, *OC* orbital contents

**Fig. 2:**
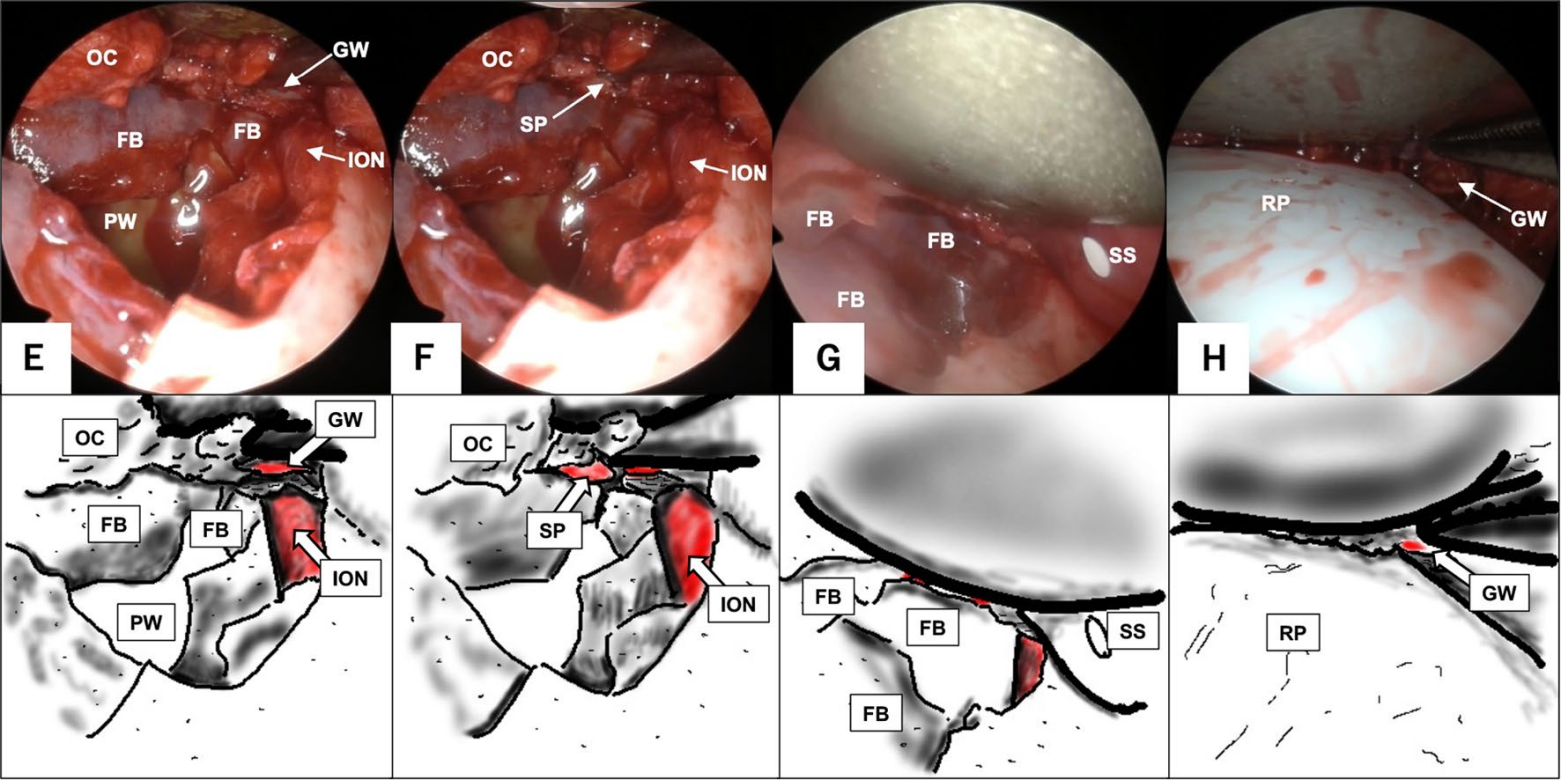
Stepwise view of the three landmarks procedure via the transorbital approach, continued from Fig. 1. Each endoscopic image is accompanied by a corresponding illustration below. The three landmarks are shown in red. The greater wing of the sphenoid bone is identified by cutting the periosteum at the dead end of the superior margin of the infraorbital nerve (**E**). After the inferior margin of the greater wing of the sphenoid bone is identified, go medial on the line of the margin. The superior posterior wall of the maxillary sinus is identified medial to the medial margin of the line (**F**). After identification of the three landmarks, orbital contents are restored with a silicone silastic sheet (**G**). The orbital floor is reconstructed with a rigid plate that is placed on the three landmarks to cover the area of the fracture. Finally, the silicone silastic sheet is removed from the orbit (**H**). *FB* fractured bone, *GW* greater wing of the sphenoid bone, *ION* infraorbital nerve, *OC* orbital contents, *PW* posterior wall of the maxillary sinus, *RP* rigid plate, *SP* superior posterior wall of the maxillary sinus, *SS* silicone silastic sheet

**Fig. 3:**
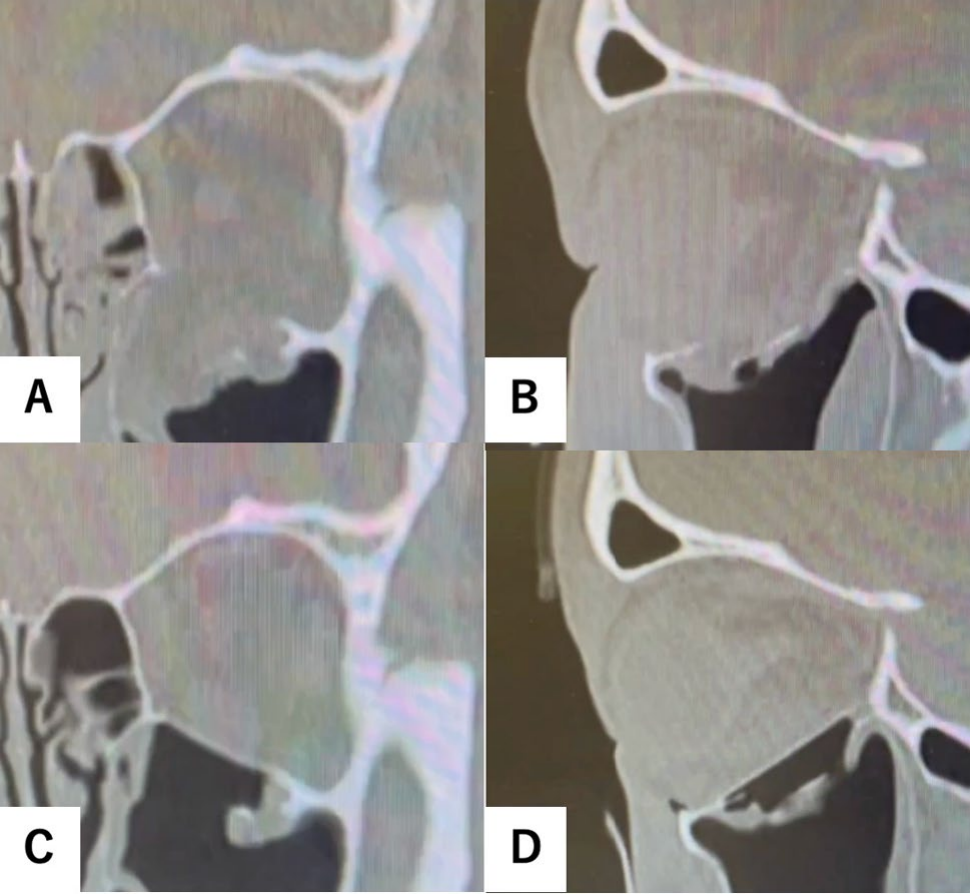
Computed tomography (CT) images before and after surgery. Images **A** and **B** are reformatted CT images before surgery. The slope of the posterior end of the orbital floor was fractured. Images **C** and **D** are reformatted CT images after surgery. The orbital contents were restored with complete reconstruction of the orbital floor, even with a fracture in the slope

TLP is a method of reconstructing the orbit that involves identifying three landmarks: the infraorbital nerve, the inferior margin of the greater wing of the sphenoid bone, and the superior posterior wall of the maxillary sinus. Although our previous report described TLP via a combined transorbital and endoscopic transnasal approach [11], it is also possible to identify and reconstruct those three landmarks using the transorbital alone (Figs. 1, 2 and 3). A poly-L-lactic acid/hydroxyapatite sheet (Super Fixorb MX® 0.3 mm sheet; Teijin Medical Technologies, Osaka, Japan) was used for reconstruction. If the fracture extended lateral to the infraorbital nerve, an additional silicone silastic sheet (Eyeball Restraint Insert; Koken, Tokyo, Japan) was implanted as needed. It was removed approximately 3 months after surgery.

#### BP

BP is a method to reduce trapped orbital contents by removing all of the fracture fragments of the orbital floor from the maxillary sinus via an endoscopic transnasal approach. The orbital contents are restored and fixed with a urethral balloon catheter (NIPRO, Osaka, Japan) placed in the maxillary sinus [6, 7]. The balloon catheter is removed 7–10 days after surgery.

### Measurements

HAR% [12] was used to measure ocular movements. It is an objective evaluation method that has been utilized in several previous reports [3, 12–14]. Enophthalmos was evaluated on the basis of the difference between the positions of the eyes, subtracting the value of the affected side from the healthy side, using a Hertel exophthalmometer [15–18].

### Outcomes

The primary outcome was the difference in postoperative ocular movements between patients who underwent TLP versus BP. Secondary outcomes were postoperative ocular movements in patients with fractures extending to the slope of the posterior end of the orbital floor and enophthalmos in patients who underwent TLP.

### Adverse events

Decrease in visual acuity, postoperative rhinosinusitis, sensory disturbance of the face, and infection of the implant materials were evaluated as adverse events.

### Data source

Clinical data were collected from chart review.

#### Bias

Selection bias and information bias were not able to be ruled out from this study.

### Statistical analysis

The Mann–Whitney U test was used to compare HAR% between the two groups. *P* < 0.05 was considered to be statistically significant. The 95% confidence interval (CI) was calculated as mean ± 1.96 times standard error. EZR [19], a freely available modified R commander, was used for all statistical analyses.

## Results

### Patients

**Fig. 4:**
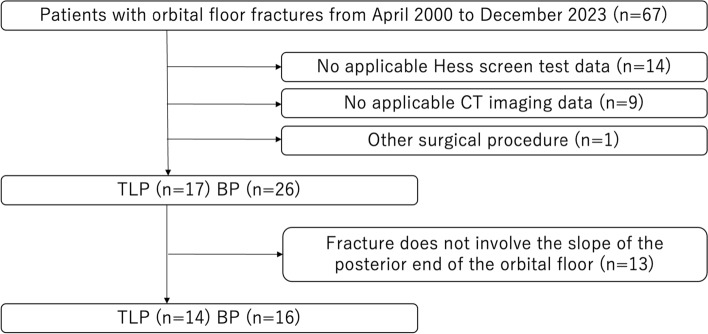
Study flow diagram. Of 67 patients, 24 were excluded because of concomitant conditions. The 43 patients included in the study consisted of 17 patients who underwent the three landmarks procedure (TLP) and 26 patients who underwent the balloon procedure (BP). Of 43 patients, 30 were included in a sub-analysis of fractures extending to the posterior end of the orbital floor. *BP* balloon procedure, *CT* computed tomography, *TLP* three landmarks procedure

During the study period, 67 patients with orbital floor fractures underwent surgery (Fig. 4). Of these, 14 patients had missing Hess screen test data, 9 patients had unusable computed tomography (CT) images, and 1 patient underwent a surgical procedure other than TLP and BP. Excluding these 24 patients, 17 patients who underwent TLP and 26 patients who underwent BP were included in this study. There were 13 patients in whom the fracture did not extend to the slope of the posterior end of the orbital floor. In the study of patients whose fractures extending to the slope, there were 14 patients in the TLP group and 16 in the BP group.

### Characteristics of the study patients

#### TLP group (Table 1)

**Table 1:** Characteristics of the patients in the TLP group (n = 17)

Characteristic	TLP group	
	Value	(%)
Sex		
Male	13	(76)
Female	4	(24)
Age, years		
Median	44	
Range	28–71	
Duration from injury to surgery, days		
Average	11.3	
Range	3–19	
Follow-up, days		
Average	711.1	
Range	56–1779	
HAR% before surgery		
Mean	72.5	
95% CI	62.3–82.7	
HAR% after surgery		
Mean	98.3	
95% CI	97.0–99.6	
Enophthalmos (healthy side—affected side), mm		
Mean	0.06	
95% CI	– 0.32 to 0.44	

*CI* confidence interval, *HAR%* percentage of Hess area ratio, *TLP* three landmarks procedure

Of the 17 patients, 13 were male and 4 were female. Median age was 44 years (range 28–71 years). Mean duration from injury to surgery was 11.3 days (range 3–19 days). Mean follow-up was 711.1 days (range 56–1,779 days). Mean HAR% before surgery was 72.5 (95% CI 62.3–82.7) and mean HAR% after surgery was 98.3 (95% CI 97.0–99.6). Mean postoperative enophthalmos was 0.06 mm (95% CI − 0.32 to 0.44 mm).

#### BP group (Table 2)

**Table 2:** Characteristics of the patients in the BP group (n = 26)

Characteristic	BP group	
	Value	(%)
Sex		
Male	19	(73)
Female	7	(27)
Age, years		
Median	30	
Range	13–78	
Duration from injury to surgery, days		
Average	13.3	
Range	2–125	
Follow-up, days		
Average	221.2	
Range	18–630	
HAR% before surgery		
Mean	68.1	
95% CI	59.9–76.3	
HAR% after surgery		
Mean	88.6	
95% CI	83.2–94.0	
Enophthalmos (healthy side—affected side), mm		
Mean	N/A	
95% CI	N/A	

*BP* balloon procedure, *CI* confidence interval, *HAR%* percentage of Hess area ratio

Of the 26 patients, 19 were male and 7 were female. Median age was 30 years (range 13–78 years). Mean duration from injury to surgery was 13.3 days (range 2–125 days). Mean follow-up was 221.2 days (range 18–630 days). Mean HAR% before surgery was 68.1 (95% CI 59.9–76.3) and mean HAR% after surgery was 88.6 (95% CI 83.2–94.0).

### Characteristics of patients with a fracture extending to the slope of the posterior end of the orbital floor

#### TLP group (Table 3)

**Table 3:** Characteristics of the patients in the TLP group with a fracture extending to the slope of the posterior end of the orbital floor (n = 14)

Characteristic	TLP group	
	Value	(%)
Sex		
Male	10	(71)
Female	4	(29)
Age, years		
Median	43	
Range	28–63	
Duration from injury to surgery, days		
Average	11.4	
Range	4–16	
Follow-up, days		
Average	704.8	
Range	56–1779	
HAR% before surgery		
Mean	71.3	
95% CI	58.9–83.7	
HAR% after surgery		
Mean	98.5	
95% CI	97.3–99.7	
Enophthalmos (healthy side—affected side), mm		
Mean	0.14	
95% CI	-0.31–0.59	

*CI* confidence interval, *HAR%* percentage of Hess area ratio, *TLP* three landmarks procedure

Of the 14 patients, 10 were male and 4 were female. Median age was 43 years (range 28–63 years). Mean duration from injury to surgery was 11.4 days (range 4–16 days). Mean follow-up was 704.8 days (range 56–1,779 days). Mean HAR% before surgery was 71.3 (95% CI 58.9–83.7) and mean HAR% after surgery was 98.5 (95% CI 97.3–99.7). Mean value of postoperative enophthalmos was 0.14 mm (95% CI − 0.31 to 0.59 mm).

BP group (Table 4).

**Table 4:** Characteristics of the patients in the BP group with a fracture extending to the slope of the posterior end of the orbital floor (n = 16)

Characteristic	BP group	
	Value	(%)
Sex		
Male	13	(81)
Female	3	(19)
Age, years		
Median	33.5	
Range	16–78	
Duration from injury to surgery, days		
Average	16.3	
Range	2–125	
Follow-up, day		
Average	214.8	
Range	26–630	
HAR% before surgery		
Mean	69.2	
95% CI	60.9–77.5	
HAR% after surgery		
Mean	89.2	
95% CI	82.4–95.8	
Enophthalmos (healthy side—affected side), mm		
Mean	N/A	
95% CI	N/A	

*BP* balloon procedure, *CI* confidence interval, *HAR%* percentage of Hess area ratio

Of the 16 patients, 13 were male and 3 were female. Median age was 33.5 years (range 16–78 years). Mean duration from injury to surgery was 16.3 days (range 2–125 days). Mean follow-up was 214.8 days (range 26–630 days). Mean HAR% before surgery was 69.2 (95% CI 60.9–77.5) and mean HAR% after surgery was 89.2 (95% CI 82.4–95.8).

### Comparison of postoperative ocular movements between the TLP and BP groups

Among all study patients, the TLP group had statistically significantly higher mean HAR% than the BP group (98.3 (95% CI 97.0–99.6) vs. 88.6 (95% CI 83.2–94.0); *p* = 0.0059). Similarly, among patients with a fracture extending to the slope of the posterior end of the orbital floor, the TLP group had statistically significantly higher mean HAR% than the BP group (98.5 (95% CI 97.3–99.7) vs. 89.2 (95% CI 82.4–95.8); *p* = 0.0122).

### Postoperative enophthalmos in the TLP group

Among all patients who underwent TLP, the mean difference in eye position between the healthy and affected side based on Hertel exophthalmometry was 0.06 mm (95% CI − 0.32 to 0.44). Among patients with a fracture extending to the slope of the posterior end of the orbital floor who underwent TLP, the mean difference was 0.14 mm (95% CI − 0.31 to 0.59). No patients had both subjective and objective enophthalmos.

### Adverse events

There were only two adverse events in the study: implant infection (n = 1) and postoperative chronic sinusitis (n = 1). One patient in the TLP group had suspected peri-implant infection and the implant was removed under general anesthesia at 1 month after primary surgery. At the time of implant removal, the strength of the reconstructed orbital bone was satisfactory and no enophthalmos occurred. One patient in the BP group had chronic sinusitis due to adhesions from the primary surgery. The patient underwent endoscopic sinus surgery under local anesthesia and completely recovered.

## Discussion

In this study, we developed TLP as a safe and reproducible technique for fractures involving the slope of the posterior end of the orbit, which is considered to be the most difficult to reconstruct [1, 2]. Results of TLP were satisfactory. The seamless recognition of three landmarks (infraorbital nerve, inferior margin of the greater wing of the sphenoid bone, and superior posterior wall of the maxillary sinus) enables reconstruction of the infraorbital wall without implants straying into the orbit or the maxillary sinus [11].

TLP significantly improved ocular movement compared with BP. In BP, the fracture fragments and maxillary sinus mucosa at the fracture site are resected. As a result, the inferior margin of the orbital contents at the fracture site is exposed in the maxillary sinus after balloon removal [6–8]. Therefore, postoperative adhesions between maxillary sinus tissue and orbital contents or slight drooping of the hummocky orbital contents may be concerns. There is less concern about adhesions and drooping with TLP relative to BP because the orbit and maxillary sinus are completely separated with implant placement.

TLP performed significantly better than BP with regard to ocular movement, even for fractures extending to the posterior slope. With previous methods, fractures extending into the slope are not stable for implant placement because the bone at the posterior margin of the fracture has collapsed, leaving the implant in a cantilevered position [1]. In TLP, the incision at the junction of the pterygopalatine fossa and infraorbital periosteum and the inferior margin of the greater wing of the sphenoid bone are identified, thereby allowing space for the implant to be placed at the posterior margin of the orbit. This allows for stabilization of the implant and complete reconstruction of the fracture site, which might prevent adhesions between orbital contents and maxillary sinus tissue, leading to a good outcome. By contrast, BP cannot completely restore orbital contents that deviate into the maxillary sinus at the posterior margin of a fracture extending to the slope due to the shape of the balloon [6–8].

None of the patients who underwent TLP had recognized postoperative enophthalmos. Previous reports have indicated that if the difference in eye position between the healthy and affected sides is 2 mm or less, the patient is not aware of enophthalmos [20–22]. Patients who underwent TLP had satisfactory results. Since rigid reconstruction is not performed in BP, enophthalmos is a concern. It has been suggested that there can be a correlation between fracture area and subjective symptoms of ocular depression [23]. Thus, reconstruction is desirable, especially if the fracture area is large [4].

In addition to the previously reported combined transorbital and transnasal approaches [11], the transorbital approach alone can also be used to perform TLP. In TLP via the transorbital approach alone, the use of an endoscope is very important for landmark identification and safe manipulation [2, 4]. Furthermore, the use of a navigation system makes the manipulation safer and more accurate [5]. The combined transorbital and transnasal approach is safer and more accurate than the transorbital approach alone because multiple surgeons can support each other in multiple directions and confirm the position of the implant [24, 25]. Although the approach used for orbital reconstruction is influenced by a surgeon’s experience, we would like to emphasize that TLP can be performed not only via the combined approach but also via the transorbital approach alone, which might be helpful for many surgeons. In addition, it should be emphasized that the endoscopic skills of otorhinolaryngologists can contribute to the treatment of the orbital floor fractures even in a multidisciplinary team with ophthalmologists and plastic surgeons.

This study has limitations. This was a single-center study. Therefore, the number of participants was small. However, a single-center study has the advantage of having a limited number of surgeons. In particular, TLP was performed by a single surgeon, thus ensuring uniformity in surgical quality. This study was not compared to conventional rigid reconstruction of the orbital floor. Nevertheless, it should be noted that mean HAR% of the TLP group was better than that in previous case series in which HAR% was measured [3, 13, 14]. Although no statistical comparisons can be made, the treatment results were satisfactory, even for fractures extending into the posterior slope. The surgeon in this study was an otorhinolaryngologist familiar with the use of endoscopes in surgery. Hence, it is unclear whether a surgeon who does not usually use endoscopes can smoothly perform this procedure. Despite these limitations, we are convinced that orbital reconstruction performed with identification of the three landmarks is a safe and reproducible procedure. The results of this study confirm the validity of this belief.

## Conclusion

TLP is a safe and reproducible technique with better results than BP. In particular, it is a technique that can successfully treat fractures extending to the slope of the posterior end of the orbital floor, which were previously thought to be difficult to repair. TLP can be performed not only in combination with the transnasal approach, but also with the transorbital approach alone, enabling TLP to support many surgeons.

## Data Availability

The data that support the findings of this study are available from the corresponding author, Y.M., upon reasonable request.
